# Mycobacterial Infections in Invasive Turtle Species in Poland

**DOI:** 10.3390/pathogens12040570

**Published:** 2023-04-07

**Authors:** Łukasz Radulski, Monika Krajewska-Wędzina, Marek Lipiec, Marcin Weiner, Anna Zabost, Ewa Augustynowicz-Kopeć

**Affiliations:** 1 County Veterinary Inspectorate in Puławy, C.K. Norwida 17, 24-100 Puławy, Poland; 2 National Veterinary Research Institute, 24-100 Puławy, Poland; 3 Department of Veterinary Microbiology, Faculty of Veterinary Medicine, Institute of Preclinical Veterinary Sciences, University of Life Sciences, 20-033 Lublin, Poland; 4 Pope John Paul II State School of Higher Education, Sidorska 95/97, 21-500 Biała Podlaska, Poland; 5 Department of Microbiology, National Tuberculosis and Lung Diseases Research Institute, 01-138 Warsaw, Poland

**Keywords:** mycobacteriosis, invasive turtle species, atypical mycobacteria

## Abstract

Over the last 30 years, the number of invasive turtle species living in the wild has significantly increased in Poland. This proliferation carries many threats, which mainly include the displacement of native species of animals from their natural habitats. Turtles can also be reservoirs for pathogens, including bacteria from the *Mycobacterium* genus. In order to confirm or rule out the presence of acid-fast mycobacteria in the population of invasive turtle species, samples from carapace, plastron, internal organs and mouth cavity swabs from 125 animals were tested. Twenty-eight mycobacterial strains were isolated in culture, which were classified as atypical following multiplex-PCR reactions. The GenoType Mycobacterium Common Mycobacteria (CM) test, matrix-assisted laser desorption/ionization time-of-flight mass spectrometry, PCR-restriction fragment length polymorphism (PRA)-*hsp65* and DNA sequencing were used to identify the species of isolates. Of the 28 strains, 11 were identified as *M. fortuitum*, 10 as *M. chelonae*, 3 as *M. avium* ssp. *avium*, 2 as *M. nonchromogenicum* and 1 each of *M. neoaurum* and *M. scrofulaceum*. The results of the research will also strengthen the understanding that these animals can be vectors for pathogens when living in the wild.

## 1. Introduction

It is estimated that there are about 12,000 alien animal species of microorganisms, fungi, plants and animals in the countries of the European Union (EU) [1]. Introducing alien animal species into the ecosystem represents the second greatest threat to biodiversity after habitat destruction [2]. In the central part of Europe, invasive species of animals often originally come from geographic regions where the predator population is much larger; therefore, these animals are often much better adapted to existence in the environment than native species [2]. In order to be ascribed invasive status, in addition to easily adapting to the new habitat, the animal species must also reproduce easily and harm the property, economy and native vegetation and animals of the region [3].

Reptiles are a small group of alien species. According to the regulations in force in Poland, four species of turtles are considered invasive: *Chrysemys picta* (*Ch. picta*), *Trachemys scripta* (*T. scripta*), *Graptemys pseudogeographica*, *Chelydra serpentine* [1,4]. The occurrence of *T. scripta* and *Ch. picta*, due to the significant damage they cause in the environment, requires preventive measures to limit their spread in Europe [5], and the introduction of these two species is prohibited in the EU [6].

*T. scripta* ornamental turtles come from the United States, where they have been intensely farmed for years and exported to many countries around the world [7]. The export volume was up to several million individuals per year, of which almost 30 percent went to Europe. In the nineties, up to 100,000 turtles of *T. scripta* subspecies were brought to Poland per year: first red-eared sliders, then yellow-bellied sliders and Cumberland sliders [8]. Turtle species that have been introduced artificially into the environment are voracious and omnivorous, thus they exploit available resources very intensively and lead to the disappearance of the population of other species. Research by Soccini et al. showed that *T. scripta* has a larger territorial range and a significant numerical advantage in Italy compared to the native *Emys orbicularis* (*E. orbicularis*) [9]. *E. orbicularis*—the European pond turtle—is under strict protection in Poland and many other European countries, and any alien species may contribute to its extinction [10].

The study results disclosed by Polish researchers indicate that *Trachemys* turtles are a source of pathogenic bacteria viruses and protozoas [11,12,13]. The red-eared slider (RES, *Trachemys scripta elegans*) ranavirus (RESRV) was isolated from a free-ranging RES turtle in eastern Poland that died with evidence of respiratory disease. The RESRV genome sequence was determined, and phylogenetic analysis revealed that it is a common midwife toad virus (CMTV) strain. This invasive chelonian represents a new host for CMTV and may potentially spread ranaviruses into native populations of fish, amphibians, and reptiles [12]. Within the same project, the presence of Cryptosporidium DNA was detected in one sample of intestinal scraping collected from a red-eared slider. A phylogenetic analysis of a 18SSU rRNA gene fragment showed 100% sequence identity between the Cryptosporidium parvum (C. parvum) strain isolated from the turtle and other C. parvum strains previously detected in cattle from the Lublin province [13]. In addition, new *Chlamydiaceae* agents have been detected in the same project. It was originally specific to freshwater turtles, and was most closely related to the well-known Chlamydia pneumoniae and the newly described *Candidatus* C. sanzinia. It was next identified in *Testudo* spp. with the highest homology to *C. pecorum* strains classified as new *Candidatus* species, and finally classified as Candidatus Chlamydia testudinis [11,14]. Different serotypes of Salmonella have been isolated, as well as many representatives of types of fungi, but these studies have not yet been published.

*T. scripta elegans* can be a source of other germs that can be transmitted not only to fish, aquatic invertebrates or birds nesting on the ground, but also to humans, especially in places that are used by people for recreation [15]. Among the bacteria whose transmission vector is turtles, we find bacteria of the genus Mycobacterium, which have zoonotic potential and are characterized by the breadth of the range of animal species that they can infect [16].

Mycobacteria are non-motile, slow-growing, rod-shaped, gram-positive and aerobic bacteria. A characteristic feature of the mycobacteria structure is their cell wall, which is much thicker than that of most other bacteria. The mycobacterial cell wall consists of lipid substances, which may constitute up to 40% of its dry weight. The cell wall of mycobacteria also includes arabinogalactan, which is unique to bacteria of the genus *Mycobacterium*. The specific composition of the cell wall and its thickness make mycobacteria extremely resistant to adverse environmental conditions [17]. These bacteria can be divided into two main groups. The first group includes tuberculosis mycobacteria, which are part of the *Mycobacterium tuberculosis* complex (MTBC), and the second group includes non-tuberculous mycobacteria (NTM). Tuberculous mycobacteria infect mammals, while atypical mycobacteria have a much broader host spectrum, which includes mammals, amphibians, reptiles, and birds. Some species of mycobacteria are responsible for causing zoonoses. There is scientific evidence that, under specific conditions, mycobacteria can be zoonotic or environmental pathogens for humans and factors that indirectly influence the development of autoimmune diseases. These diseases are transmitted by animals, and human infection usually occurs through direct contact with the carrier or raw materials of animal origin [18]. The immunomodulatory capacity of these bacteria has been known for over 50 years thanks to numerous published research works. It has been shown that certain chemical products produced by mycobacterial cells participate in inflammatory pathways involving the pathogenesis of important human diseases such as Crohn’s disease, asthma, psoriasis, osteoarthritis, Blau syndrome, sarcoidosis and autism [19]. Mycobacteria can influence inflammatory pathways not only as living organisms, but also with components derived from dead cells. The immunomodulatory potential of mycobacteria results mainly from the specific structure of their cell wall [8]. Mycobacteria can be found in large numbers in watercourses that collect water from pastures, in river and lake sediments, and in soil. The hydrophobic nature of the mycobacterial cell wall is also responsible for the bacteria’s easy aerosolization. This feature greatly facilitates their spread in the environment and is one of the factors responsible for the frequent occurrence of mycobacterial infections among free-living animals. The second feature important for the spread of these microorganisms is their high resistance to environmental conditions. Under favorable conditions, bacteria of the genus *Mycobacterium* can survive in the environment for several years while maintaining their pathogenic features [17,19].

The present survey is part of a grant project titled “Invasive turtle species as a source and vector of animal and human pathogens” that was created to assess environmental and epidemiological threats posed by invasive alien species turtles and their impact on biodiversity in Poland. This interdisciplinary project included trapping, clinical examination, quarantine, euthanasia of individuals in poor physical condition and their anatomopathological examination. The aim of our study was to confirm or rule out the infection of free-living animals with bacteria belonging to the genus *Mycobacterium* and to determine the species of the isolated strains.

## 2. Materials and Methods

### 2.1. Sample Collection

**Figure 1:**
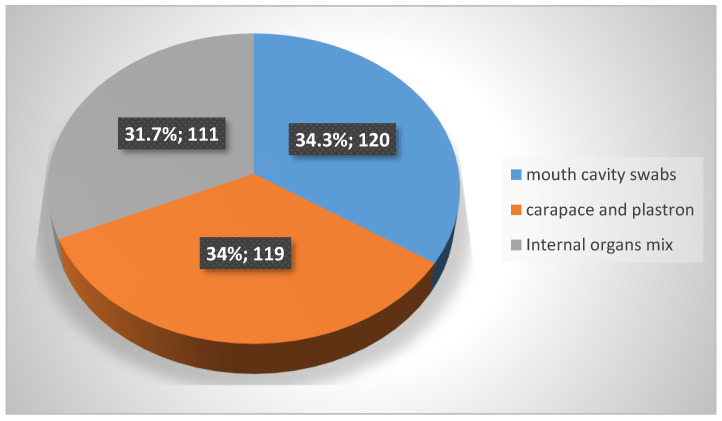
Type and number of samples taken from 125 tested turtles.

The present survey is part of a project titled “Invasive turtle species as a source and vector of animal and human pathogens” that was created to assess environmental and epidemiological threats posed by IAS turtles and their impact on biodiversity in Poland. The study covered the tissue material of turtles caught in traps located within selected reservoirs, rivers and canals located mainly in the Lublin voivodeship. Euthanized animals were subjected to health evaluation and subsequent post-mortem examination to obtain tissue samples. From 125 turtles belonging to 9 species (Table 1), samples from carapace, plastron, internal organs (liver, spleen, kidneys) and mouth cavity swabs were collected. A total of 350 samples were tested, as illustrated in Figure 1. Samples were preserved in sterile packages, which were labeled with the sample type and animal ID. The test was carried out within 24 h from the moment of sampling.

### 2.2. Mycobacteral Isolation

A 15 mL volume of 5% oxalic acid was added to approximately 3 g of finely cut tissue with a sterile scalpel, placed in a sterile filter bag and the mixture was then homogenized for 3 min in MiniMix tissue homogenizer (Interscience, Saint-Nom-la-Bretèche, France). The filtrate was poured into a Falcon tube and incubated at 37 °C for 20 min to increase the decontamination efficiency of the test sample. After incubation, the sample was centrifuged at 4000 rpm for 10 min, then washed with 15 mL of sterile physiological saline and centrifuged again at 4000 rpm for 10 min. The rinsing step was performed twice and whole supernatant was subsequently removed. The prepared material in the volume of one inoculation loop calibrated on 10 µL was plated on 6 Stonebrink and 6 Petragnani solid media, and then divided into two groups: one incubated at 37 °C ± 2 °C and the other at 25 °C ± 1 °C. An amount of 10 µL of the material was also inoculated at 37 °C on 7H9 agar liquid media using the Bactec mycobacterial growth indicator tube (MGIT) system (Becton Dickinson, Franklin Lakes, NJ, USA) and 0.5% N-acetyl-1-cysteine as a decontamination agent [20].

### 2.3. Strain Inactivation

Mycobacteria are pathogenic microorganisms to humans; therefore, they should be killed before their nucleic acids are isolated. The critical point of any molecular method is therefore inactivating the sample while minimizing the impact of inactivation on the quality of the extracted DNA. To this end, a thermal method was used. An amount of material corresponding to six volumes of an inoculation loop calibrated at 1 µL was transferred to an Eppendorf screw-cap tube containing 100 µL of sterile distilled water. In the case of liquid media, the contents of the tube were centrifuged and the sediment was suspended in 100 µL of sterile distilled water and subsequently transferred to a 2 mL screw-cap tube. The tubes were then placed in a 100 °C heated thermoblock and incubated for 10 min.

### 2.4. DNA Extraction

The Genomic Mini AX Bacteria kit (A&A Biotechnology, Gdynia, Poland) with gravity-operated DNA purification columns was used for the extraction of nucleic acids according to the protocol provided by the manufacturer of the kit, which allowed for the extraction of high-quality nucleic acids. DNA concentration was tested using a nanodrop device (Thermo Fisher, Waltham, MA, USA) following the manufacturer’s instructions. After verifying the degree of DNA contamination with proteins and chemical compounds, the degree of DNA fragmentation was checked by performing electrophoresis on a 2% agarose gel (90 V, 70 min). The appearance of a dense band indicated the lack of fragmentation of deoxyribonucleic acid, while fragmented DNA appeared in the form of a characteristic streak consisting of molecules of various sizes. The extracted nucleic acids were used both in multiplex PCR and in next-generation sequencing (NGS).

### 2.5. Multiplex PCR

To determine the type of mycobacteria (NTM or MTBC), a multiplex PCR was performed in a Biometra T1 Thermocycler (Biometra, Göttingen, Germany) with the following cycle parameters: an initial denaturing cycle for 10 min at 95 °C, then 35 cycles of 30 s each at 96 °C, followed by an annealing cycle for 2 min at 65 °C, an extension cycle for 3 min at 72 °C, and a final extension cycle for 10 min at 72 °C. The products of the PCR were stored at 4 °C until further use. Two pairs of primers were used in this reaction. The first of them, forward 5′-AGA GTT TGA TCC TGG CTC AG-3′ and reverse 5′-TGC ACA CAG GCC ACA AGG GA-3′, was responsible for the amplification of a 1030 bp fragment of DNA encoding 16 S rRNA unique to each species belonging to the genus *Mycobacterium*. The second pair of primers, forward 5′-GAA CAA TCC GGA GTT GAC AA-3′ and reverse 5′-AGC ACG CTG TCA ATC ATG TA-3′, was responsible for the amplification of 372 bp of the DNA fragment located within the MPB 70 gene, whose presence in the genome is characteristic only of mycobacteria in the MTBC. The final stage of the study was a 2% agarose gel electrophoresis (70 V, 80 min) in 1× TBE buffer. Simply Safe (EURx, Gdansk, Poland) was used as the DNA visualization dye. The appearance of the reaction product with a length of 1030 bp indicated that the tested strain belonged to the bacteria of the genus *Mycobacterium*. The presence of a second reaction product with a length of 372 bp indicated that the analyzed strain belonged to the *Mycobacterium tuberculosis* complex. The results of the multiplex PCR test established how the appropriate species identification methods were selected because these methods differ depending on the type of mycobacteria tested for.

### 2.6. Strain Species Identification

The species identification process of the isolated strains was carried out using 4 methods. The laboratory implemented new species identification methods in the second half of 2017 (GenoType CM, MALDI-TOF, NGS). The study of 6 strains isolated in 2016 was performed using GenoType CM, GenoType AS and the currently unused PRA *hsp*65 method.

The GenoType Mycobacterium Common Mycobacteria (CM) test (Hain Lifescience, Nehren, Germany) and Geno Type Mycobacterium AS (“Additional Species”) was used for species identification, following the manufacturer’s instructions. This test is qualitative and is designed for in vitro diagnosis of atypical mycobacteria. It is based on a PCR technique that targets the unique sequences of the 23S rRNA gene. The test uses test strips coated with specific probes complementary to the amplified nucleic acids. After chemical denaturation, single-stranded amplicons bind to the probes and hybridization occurs. On each strip, there are 14 areas (GenoType CM), or 19 (GenoType AS) of specific DNA characteristic for each NTM species and the three controls. The color of the strip indicates the presence of a DNA sequence characteristic for a given bacterium in the tested sample [21].

The method used to confirm the results of the Hain Lifescience CM test was matrix-assisted laser desorption/ionization time-of-flight (MALDI-TOF) mass spectrometry with a Bruker Biotyper System (Bruker, Billerica, MA, USA) and NCBI (National Center for Biotechnology Information) as the reference database. For this purpose, an amount of bacterial biomass corresponding to 2 volumes of an inoculation loop calibrated at 1 µL was added to a test tube containing 50 µL of trifluoroacetic acid. The prepared sample was incubated for 30 min at room temperature, and was then diluted tenfold by adding 450 µL of sterile distilled water. Afterwards, 1 µL of the diluted mixture was taken and applied to a MALDI plate. After the applied sample was dry, 1 µL of the matrix was added and analyzed.

Identification of 3 out of the 6 tested strains from the year 2016 was achieved by PCR-restriction fragment length polymorphism (RFLP) analysis of the heat shock protein hsp65 gene. A fragment of the DNA encoding the heat shock protein was amplified by two specific primers (Tb11 [5′-ACCAACGATGGTGTGTCCAT] and Tb12 [5′-CTTGTCGAACCGCATACCCT]), as described by Brunello et al. [22]. Separate aliquots of the PCR product were digested with BstEII for 2 h at 60 °C and HaeIII for 2 h at 37 °C, and the resulting restriction fragments were separated by electrophoresis in a 3% agarose gel (120 V, 2 h) with 50 and 25 bp ladder as molecular size standard and read.

In order to identify the subspecies of the strains referred to as *M. avium* and the mycobacterial species that could not be identified using the other methods, NGS was performed in order to enable whole genome sequencing. After quality control of the extracted DNA, DNA libraries were prepared. The Nextera XT DNA Library Preparation Kit (Illumina, San Diego, CA, USA) was used for this purpose according manufacturer instructions. The samples were sequenced on the Illumina MiSeq platform using the MiSeq Reagent Kit v3 sequencing kit (Illumina, San Diego, CA, USA). The sequences IS901, IS1245 and IS311 were present in the genome of all representatives of the *M. avium* species and were used to identify the subspecies. However, in order to identify the species of the currently unidentified strain, the analysis of the coding sequence of the 16SrRNA gene, which was present in the genome of all bacteria of the genus Mycobacterium, was used. For this purpose, the NCBI BLAST program was used, in which the obtained sequences were compared with all sequences available in the GenBank database. A strain was considered identified if the same result was obtained in the analysis of each sequence, characterized by 100% sequence similarity coverage with the test sequence.

## 3. Results

From the total of 125 animals sampled, 28 had plastron and carapace lesions. The main lesions on them were small pits on its surface with discoloration of the covering scales (both darkening and discoloration). Exfoliation and slight protrusion from the surface were observed in both (plastrons and carapaces). In the place of the ongoing scales process, they could be removed relatively easily.

Thirty-one were positive for bacteria from the genus *Mycobacterium*, which represents a total prevalence of 24.8%. This result was confirmed by cell culture. All plastron and carapace lesions were correlated with mycobacteria isolation. The largest number of positive samples came from the central part of the Lublin voivodeship, where turtles were caught near the Zemborzycki Lake.

### 3.1. Multiplex PCR

**Figure 2:**
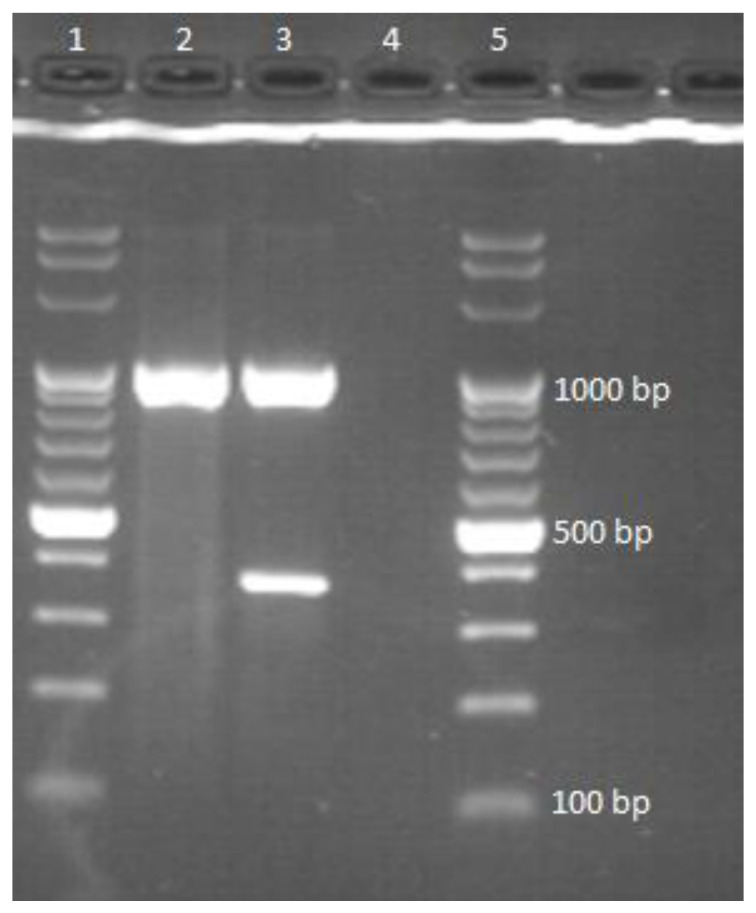
Interpretation of Multiplex PCR results: lanes 1, 5—DNA ladder, 2—pattern for nontuberculous mycobacteria; 3—pattern for MTBC, 4—pattern for species other than mycobacteria.

All 28 strains were classified as atypical mycobacteria. Visualization of the PCR results showed that in each case, only the DNA fragment that was part of the gene encoding the 16 S rRNA was amplified. Based on the results of the multiplex PCR reaction, the Hain Lifescience CM MALDI-TOF species identification method was selected for each tested strain; in the case of *M. avium* isolation, DNA sequencing was the appointed method. Figure 2 shows an example of result visualization. Each tested sample showed only one distinct DNA band, which clearly indicated that the tested strain was an atypical *Mycobacterium* sp.

### 3.2. Hain Lifescience CM

**Figure 3:**
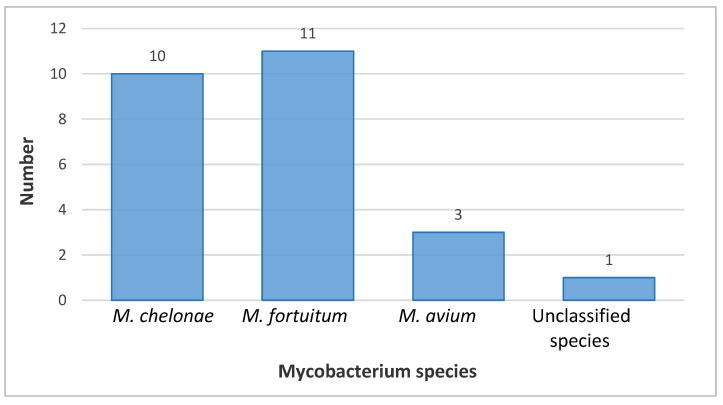
Hain Lifescience Common Mycobacteria (CM) Test identification of Mycobacteria species.

Using the Hain Lifescience CM test, 24 out of 25 strains (96%) were clearly identified. In one case, an ambiguous result was obtained, indicating that the identified mycobacterium was either *M. scrofulaceum* or *M. intracellulare*, which may be due to the high genetic similarity of these two species [23]. The most common pathogens responsible for the infection/contamination of turtles were *M. chelonae* and *M. fortuitum*, as shown in Figure 3.

### 3.3. MALDI-TOF

**Figure 4:**
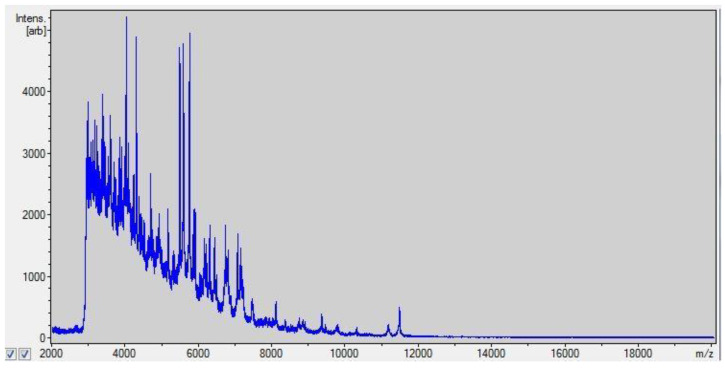
Matrix-assisted laser desorption/ionization time-of-flight spectrum for *M. chelonae*.

Twenty-two strains of NTM were tested. Mass spectrometry and the Hain Lifescience tests identified the species of 21 out of 22 strains tested (95.45%). Figure 4 shows an example reading of the spectrum for *M. chelonae.*

### 3.4. RFLP-PRA hsp65

**Figure 5:**
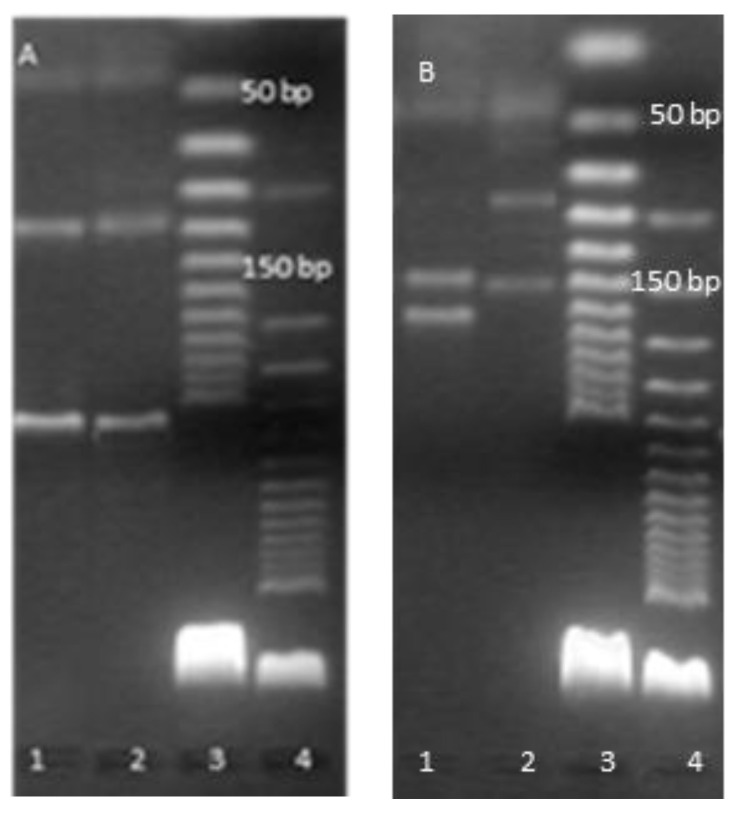
PCR restriction enzyme pattern analysis of the hsp65 gene: (**A**) PCR product were digested with BstEII line 1—pattern for *M.neoaurum*, 2—pattern for *M.nonchromogenicum,* 3—DNA ladder 25 bp, 4—DNA ladder 50 bp; (**B**) PCR products were digested with HaeIII line 1—pattern for *M.neoaurum*, 2—pattern for *M.nonchromogenicum,* 3—DNA ladder 25 bp, 4—DNA ladder 50 bp.

Analysis was performed for three strains that were not identified with species by GenoType CM and GenoType AS tests. The following NTM species were identified: *M. nonchromogenicum* (*n* = 2) and *M. neoaurum* (*n* = 1).(Figure 5).

### 3.5. DNA Sequencing

DNA sequence analysis was performed on three strains of *M. avium* and a strain that failed to be identified using other methods. In each case analyzed, the subspecies was established as *M. avium* ssp. *avium*. A mycobacterial species that was not identified using the Hain Lifescience CM or MALDI-TOF assays was *M. scrofulaceum*.

### 3.6. Summary of the Results

A total of 28 strains of atypical mycobacteria belonging to 6 different species were isolated from 31 turtles. Most cases of infection/contamination with bacteria from the genus *Mycobacterium* were noted in red-eared sliders, and *M. chelonae* was the most frequently isolated mycobacterium species. One of the red-eared sliders was found to be co-infected with *M. fortuitum* and *M. nonchromogenicum*. The results are summarized in Table 2. Mycobacteria were most often isolated from carapace and plastron samples (*n* = 24), and less often from mix of internal organs (*n* = 8) and mouth cavity swabs (*n* = 4). In three cases, positive results were obtained from two types of tested samples, and in one case from all three. 

## 4. Discussion

The high number of infections/contaminations with bacteria from the genus *Mycobacterium* among the examined turtles and the shared etiology of those infections among as many as six species of mycobacteria may indicate the high susceptibility of turtles to infections with these pathogens. In contrast, the strains’ isolation from at least two different samples from the same animal in only four cases and the infections of internal organs seen in only eight cases may prove that the disease was often absent and the presence of mycobacteria in the sample was due to their absorption by turtles (in regard to mouth swabs) and to the adhesion of the bacilli to their carapaces.

Mycobacteria are often found in water [24]. Aquatic animals, such as turtles, which are a vector of mycobacterial transmission, can also be a threat to humans. Unaware of the danger, a person may capture a free-living turtle and keep it as a pet. Contact with aquarium water can result in human infection, which has already been documented [25]. Fish can also be a vector for transmission of the same mycobacterial species as turtles. The literature describes cases of isolation of *M. fortuitum* from aquarium fish that were randomly collected for research from four different aquariums [26]. *Mycobacterium chelonae* is also a microbe that is often responsible for causing mycobacteriosis in aquarium fish. Mycobacteriosis is the second most common infectious disease in zebrafish research colonies, and most often this is caused by *M. chelonae*. The infection is characterized by multiple granulomas in the kidney and coelomic cavity, and particularly in the ovary [27].

*Mycobacterium chelonae* was isolated from 10 tested animals. Seven out of ten individuals showed skin, carapace and plastron lesions, including pigmentation defects, deformities, local inflammation and abrasions. Very often, these symptoms were also accompanied by low activity of the animal resulting from its weakening by the infection. A similar course of *M. chelonae* mycobacteriosis was observed by Murray et al. [28]. In *Apalone spinifera*, the spiny softshell turtle, the disease manifested as a local lack of pigmentation of the plastron and lethargy. Additionally, there was bleeding from the nostrils and ecchymosis on the limbs. This species of *Mycobacterium* can also infect sea turtles. An example of this is the *Lepidochelys kempii* (Kemp’s ridley sea turtle) mycobacteosis found by Greer et al. [29]. Similar to the previously described cases, the affected animal had lesions of the plastron. After four months, limb edema appeared, from which scientists cultured pure *M. chelonae*.

The most frequently isolated species was *M. fortuitum*, which was confirmed as being present in 14 individuals. The lesions noted in *M. fortuitum* infections were of the skin and abnormalities in the structure of the carapace and plastron, as in *M. chelonae* mycobacteriosis. Necrotic tissue on the pelvic limbs was also noted in one red-eared slider. Clinical cases in reptiles associated with these mycobacteria are not reported in the literature. In fact, this species has been often isolated from fish with or without macroscopic lesions. This high prevalence is probably related to the common presence of *M. fortuitum* in the aquatic environment, where its presence is strongly related to management factors such as the quality and quantity of nutrients, water supply and temperature [30].

*Mycobacterium avium* is a mycobacterial species that is rarely responsible for mycobacteriosis in reptiles. However, this was isolated from three examined turtles and the subspecies were identified as *M. avium* ssp. *avium* by DNA sequencing. This *Mycobacterium* sp. is most often responsible for causing disease in birds; therefore, it can be considered that the probable source of infection was waterfowl. The examined animals did not show any serious symptoms; two of them presented abnormalities, which were deformities of the carapace. However, these may have been related to a poor diet. In the only documented case of turtle mycobacteriosis caused by *M. avium*, Brock et al. described the disease in six representatives of the green turtle (*Chelonia mydas*) [31]. The course of the infection was acute, which resulted in the formation of multiple organ lesions. Establishing a pattern of infection development with a small number of tested animals is therefore quite a challenge. The absence of visible disease symptoms may indicate that turtles are often asymptomatic carriers of *M. avium*. Being in an aquatic environment, which is often also a bird habitat, significantly increases the risk of getting bacteria into the digestive tract or deposited on the surface of the carapace and skin. The accidental deposition of bacteria on the animal’s carapace was also the likely reason why *M. scrofulaceum* could be isolated from the red-eared slider. Until now, reptile mycobacteriosis caused by this mycobacterial species has not been described. A similar puzzle is also why *M. neoaurum* could be isolated from internal organs and the carapace and plastron of one of the red-eared sliders. There were lesions on the animal’s body in the area of the eyes, skin and scutum. No clinical case of turtle mycobacteriosis caused by this pathogen has been reported in the literature so far, and *M. neoaurum* is most often responsible for human rather than animal diseases [32].

*Mycobacterium nonchromogenicum* is a mycobacterium that is often relatively responsible for mycobacteriosis in reptiles [33]. This bacterium is often isolated from soil samples and is therefore widespread in the environment [34]. This *Mycobacterium* sp. in turtles can cause both skin lesions on the limbs and nodular changes in internal organs [33]. In each of the three analyzed cases, *M. nonchromogenicum* was isolated only from the internal organs of animals, which excludes accidental adhesion of mycobacteria to the shells of turtles. The first of the turtles infected with *M. nonchromogenicum* developed a marked deformation of the carapace. In the plastron of the second turtle, in the right thoracic plate, minor necrotic foci and extravasation on the abdominal plastron plates were noted. The lesions slightly resembled those described by Muro et al., who isolated *M. nonchromogenicum* from six Hermann’s tortoises (*Testudo hermanni*) [35]. After the post-hibernation period, the animals were depressed and began to lose weight. In addition, inflammation and edema were observed in the distal joints of the hind limbs of four animals, and inflammation and edema were observed in the tails of two animals. The species *M. nonchromogenicum* is considered non-pathogenic for humans; however, it can cause human disease, albeit rarely. Pulmonary disease of this etiology is extremely rare, but such cases have been reported in the literature [36].

## 5. Conclusions

The aim of the study was to exclude or confirm the infection of invasive turtle species living in Poland through bacteria of the genus *Mycobacterium*. The test results clearly showed that the examined animals were infected or contaminated with NTM pathogens. Most of these pathogens are often responsible for diseases, as well as in many species of domestic, farm and wild animals. The results of the research will contribute to the understanding that these animals living in the wild can be reservoirs for pathogens.

## Figures and Tables

**Table 1:** Number of tested animals and tissue lesions.

No.	Turtle Species	Number of Individuals	Individuals with Plastron and Carapace Lesions
1	*Trachemys scripta elegans*, red-eared slider	73	21
2	*Trachemys scripta scripta*, yellow-bellied slider	32	4
3	*Trachemys scripta troostii*, Cumberland slider	12	0
4	*Pseudemys concinna*, river cooter	2	2
5	*Graptemys pseudogeographica*, false map turtle	2	0
6	*Chelydra serpentine*, common snapping turtle	1	0
7	*Testudo Hermanni*, Hermann’s tortoise	1	0
8	*Pseudemys nelsoni*, Florida redbelly turtle	1	1
9	*Mauremys sinensis*, Chinese stripe-necked turtle	1	0
Total		125	28

**Table 2:** Cumulative results of all identified strains.

No.	Turtle Species	Species of the Isolated Mycobacterium	Number of Strains	Number of Infected/Contamined Animals
1.	*Trachemys scripta elegans*	*M. chelonae*	4	17
		*M. fortuitum*	8	
		*M. nonchromogenicum*	1	
		*M. neoaurum*	1	
		*M. avium ssp. avium*	2	
		*M. scrofulaceum*	1	
2.	*Trachemys scripta scripta*	*M. chelonae*	5	7
		*M. fortuitum*	2	
3.	*Trachemys scripta troostii*	*M. fortuitum*	1	3
		*M. avium ssp. avium*	1	
		*M. nonchromogenicum*	1	
4.	*Pseudemys concinna*	*M. chelonae*	1	1
Total			28	28

## Data Availability

No new data were created or analyzed in this study. Data sharing is not applicable to this article.
